# Reassessing the Risk of Severe Parvovirus B19 Infection in the Immunocompetent Population: A Call for Vigilance in the Wake of Resurgence

**DOI:** 10.3390/v16091352

**Published:** 2024-08-24

**Authors:** Giancarlo Ceccarelli, Francesco Branda, Alessandra Ciccozzi, Chiara Romano, Daria Sanna, Marco Casu, Mattia Albanese, Francesco Alessandri, Gabriella d’Ettorre, Massimo Ciccozzi, Fabio Scarpa, Marta Giovanetti

**Affiliations:** 1 Department of Public Health and Infectious Diseases, University of Rome Sapienza, Rome, Italy and Azienda Ospedaliero Universitaria Policlinico Umberto I, 00161 Rome, Italy; dott.albanese.mattia@gmail.com (M.A.); gabriella.dettorre@uniroma1.it (G.d.); 2 Unit of Medical Statistics and Molecular Epidemiology, Università Campus Bio-Medico di Roma, 00128 Rome, Italy; f.branda@unicampus.it (F.B.); chiara.romano@unicampus.it (C.R.); m.ciccozzi@unicampus.it (M.C.); 3 Department of Biomedical Sciences, University of Sassari, 07100 Sassari, Italy; aciccozzi@uniss.it (A.C.); darsanna@uniss.it (D.S.); fscarpa@uniss.it (F.S.); 4 Department of Veterinary Medicine, University of Sassari, 07100 Sassari, Italy; marcasu@uniss.it; 5 Department of General and Specialistic Surgery, Sapienza University of Rome, 00161 Rome, Italy; francesco.alessandri@uniroma1.it; 6 Department of Sciences and Technologies for Sustainable Development and One Health, Università Campus Bio-Medico di Roma, 00128 Rome, Italy; giovanetti.marta@gmail.com; 7 Instituto René Rachou, Fundação Oswaldo Cruz, Belo Horizonte 30190-002, MG, Brazil; 8 Climate Amplified Diseases and Epidemics (CLIMADE), Brasilia 70070-130, GO, Brazil

**Keywords:** Parvovirus B19, outbreaks, viral monitoring, public health

## Abstract

Despite Parvovirus B19 (B19V) generally causing mild or asymptomatic infections, and only certain high-risk groups such as hematological or immunocompromised patients and pregnant women tending to develop complications, several factors challenge the assumption of a “benign” clinical course in immunocompetent adults and adolescents. A significant proportion of the population may harbor undiagnosed health conditions or genetic predispositions that could render them more susceptible to severe B19V complications. These could include mild hematological disorders, immune dysregulation not resulting in overt immunodeficiency, or underlying cardiac conditions. Concurrent infections with other pathogens, even seemingly minor ones, could synergistically increase the severity of B19V infection, leading to more pronounced clinical manifestations. While not definitively proven, the possibility of emerging B19V strains with increased virulence or altered tissue tropism cannot be entirely discounted. Additionally, the period of pandemic-related restrictions likely led to reduced B19V circulation, potentially resulting in a cohort of young adults with limited natural immunity, making them more vulnerable to infection. Potential clinical consequences include atypical and severe presentations, even in individuals without known risk factors. The traditional focus on B19V primarily as a pediatric concern might lead to underdiagnosis or delayed diagnosis in adults, potentially hindering timely intervention and management. A surge in B19V-related complications, even if individually mild, could collectively strain healthcare resources, particularly in settings with limited capacity or pre-existing pressures. Possible recommendations are to heighten clinical awareness with a high index of suspicion for B19V infection in adults and adolescents presenting with compatible symptoms, even in the absence of classic risk factors. Additionally, expanding testing criteria and enhancing public health surveillance efforts would be prudent.

## 1. Introduction

Parvovirus B19 (B19V) is a ubiquitous human virus, known for its ability to cause a range of clinical presentations, from the typically mild “fifth disease” in children to more serious complications in certain individuals [1,2,3]. While an infection often goes unnoticed or results in self-limiting illness, B19V can pose significant health risks to specific populations, including pregnant women, individuals with compromised immune systems, and those with certain hematologic disorders [4,5,6,7]. This seemingly innocuous virus, with its single-stranded DNA genome, has evolved sophisticated mechanisms to evade the immune system and persist within the human population. Its predilection for infecting and disrupting red blood cell precursors underpins the diverse clinical manifestations of B19V infection [1].

While historically considered a mild childhood illness, the recent resurgence of B19V infections in Northern Europe raises concerns about its potential impact on seemingly healthy adults and adolescents [8,9,10]. The changing dynamics of B19V infection necessitate a shift in perspective, moving away from the assumption of universal benignity in immunocompetent individuals. A proactive and vigilant approach, encompassing heightened clinical awareness, broader testing strategies, and robust surveillance, is essential to mitigate the potential clinical impact of this resurgent pathogen.

Understanding the epidemiology, transmission dynamics, and potential complications of B19V is crucial for effective public health measures, timely diagnosis, and appropriate clinical management. This is particularly relevant in the context of recent viral resurgence and evolving herd immunity patterns, which necessitate a reassessment of traditional risk stratification and clinical vigilance. In this narrative review, we analyzed the new insight of B19V, focusing on epidemiology and clinical presentations.

## 2. B19V, an Old Virus: What is New?

**Figure 1:**
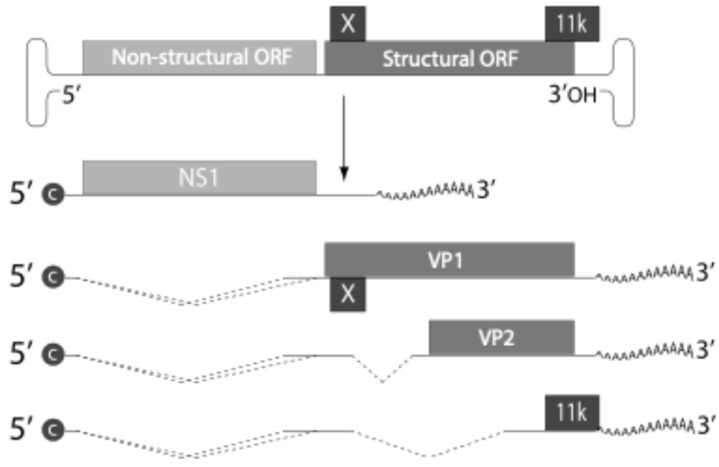
Representation of the genomic structure of the B19V genome (ViralZone https://viralzone.expasy.org, accessed on 1 Augst 2024).

B19V, a small, non-enveloped DNA virus within the *Parvoviridae* family (genus Erythrovirus), is primarily transmitted through respiratory routes and is endemic worldwide. The B19V genome is a single-stranded DNA containing 5596 nucleotides (nt), composed of an internal coding sequence of 4830 nt flanked by terminal repeat sequences of 383 nt each (Figure 1) [1].

From a phylogenetic perspective, recent research has made the noteworthy observation that contemporary genotypes of B19V have been identified in ancient human remains, indicating that these viruses have undergone minimal genetic changes over the past 4500 to 6000 years [11,12]. The genome of B19V consists of the following two primary open reading frames (ORFs): one on the left side encoding the nonstructural protein NS1, and another on the right side encoding the structural proteins VP1 and VP2 [13]. The transcriptional activity of B19V generates several overlapping mRNA transcripts, with the most significant viral proteins being NS1, VP1, and VP2 [14].

In the figure, these ORFs are depicted with the NS1 ORF on the left, responsible for encoding the nonstructural protein, while the right ORF encodes the structural proteins, VP1 and VP2. Notably, the VP1 protein includes additional amino acids at its amino terminus, distinguishing it from VP2. The figure also highlights the presence of a small protein labeled “X,” involved in the lesser-understood viral functions. Sequence variability in B19V has been observed, particularly in the VP1 and VP2 regions, with up to 3% variability at the amino acid level. Despite this, there is no evidence of more than one antigenic strain, and the virus maintains a high degree of homology across different isolates [15,16]. The figure provides a structural overview of these genomic elements, reflecting the transcriptional and translational processes that generate the key viral proteins. B19V is classified into three genotypes—1, 2, and 3—showing genetic divergence of between 2% and 13%. Genotype 1 is the most prevalent, with genotypes 2 and 3 occurring less frequently. However, no significant clinical differences have been associated with these genotypes [17].

## 3. A New Epidemiology for Parvovirus B19

B19V is primarily transmitted through respiratory routes and is endemic worldwide. B19V is not a notifiable disease, and its symptoms are typically mild, often obviating the need for laboratory diagnosis. Consequently, laboratory-confirmed infections represent only a fraction of all B19V cases [18]. Historically, recurrent epidemics of childhood diseases such as measles, mumps, and whooping cough exhibited annual patterns and more significant epidemics every two to five years, driven by the gradual build-up of susceptible populations due to births [19]. This dynamic also applies to B19V, which has demonstrated one- and four-year cyclical patterns in Europe and Australia [18]. In Europe, B19V infections predominantly occur during the spring and early summer, with outbreaks of varying intensity observed over several years [20]. During the COVID-19 pandemic, various non-pharmaceutical interventions were implemented to curb SARS-CoV-2 transmission, including lockdowns, enhanced hand hygiene, social distancing, and mask-wearing [8,9,10,21]. These measures effectively reduced the transmission of respiratory viruses like influenza and respiratory syncytial virus (RSV) [21]. Consequently, B19V infections were nearly absent from 2020 to 2022, with only low-level infections reported by the Sentinel Surveillance system. This period saw a disruption in the typical annual B19V epidemic cycle, which was absent for three consecutive years. Observational data indicate that the epidemiology of respiratory-transmitted viruses was differentially impacted by COVID-19. For instance, RSV epidemics were delayed by about six months, while influenza followed its regular epidemic pattern with flattened peaks post-2020 [22].

The decrease in B19V DNA-positive blood donation pools in France from March 2020 to November 2022 corresponded with the enforcement of COVID-19 control measures. A comparable trend was observed in other European countries [23]. From the summer of 2021 onwards, as COVID-19 restrictions in France were relaxed—including the cessation of mask recommendations in June 2021 and the reduced adherence to hygiene practices by 2022—there was an increase in B19V DNA-positive pools in 2023, with rates returning to pre-COVID-19 levels.

B19V differs from other respiratory transmitted viruses by conferring long-lasting immunity, reducing the population’s susceptibility over time. The reintroduction of B19V might occur through transcontinental transmission, necessitating the monitoring of genotype distribution upon resurgence. Globally, genotype 1 is prevalent in Europe and North America, while genotype 3 is common in Africa [24,25].

Most EU/EEA countries have not yet established a surveillance system for B19V infections. For this reason, the European Centre for Disease Prevention and Control (ECDC) has requested any available epidemiological information in order to assess the situation. This request is managed through the European surveillance portal for infectious diseases (EpiPulse), an online portal for European public health authorities and partner organizations, which facilitates the collection, analysis, sharing, and discussion of infectious disease data for threat detection, monitoring, risk assessment, and outbreak management. 

Since late 2023, numerous European countries have reported an increase in B19V infections among children, pregnant women, and blood donors [10]. From 2015 to early 2024 in France, out of the 270,508 samples analyzed (corresponding to 25,968,768 donations), 1863 (0.69%) were positive for B19V DNA. Three periods can be distinguished as follows: the pre-COVID-19 period (P1: 2015–2019) with a prevalence of 0.63% (95% CI: 0.59–0.68), the COVID-19 period (P2: 2020–2022) with a prevalence of 0.07% (95% CI: 0.05–0.09), and the post-COVID-19 period (P3: January 2023-March 2024) with a prevalence of 2.40% (95% CI: 2.24–2.56). The differences in prevalence between the P1 and P2, P3 and P2, and P3 and P1 periods were statistically significant (chi-squared test: *p* < 0.0001) [23].

In Denmark, about 60% of adults are immune to B19. Parvovirus B19 infections are endemic year-round with epidemics every 3–4 years, especially in spring. During epidemics, 13% of seronegative pregnant women are infected, compared with 1.5% during nonepidemic periods. As highlighted in the latest bulletin from the Statens Serum Institut [26], a marked seasonal cyclicality is evident, with recurrent peaks tending to occur in the spring and summer months. Specifically, in 2017, there was a significant peak with 671 cases, including 102 pregnant women, 15 of whom required hospitalization. The post-2019 period showed a significant decrease in both the number of cases and positivity rate, likely influenced by the COVID-19 pandemic and related containment measures. However, in late 2023 and early 2024, a sharp increase was observed, suggesting a possible return to pre-pandemic patterns. Interestingly, the number of Polymerase Chain Reaction (PCR)-positive cases was generally lower than Immunoglobulin M (IgM)-positive cases, yet the PCR test often exhibited a higher positivity rate. This discrepancy aligns with the criteria outlined in Table 1, where PCR is noted for its higher sensitivity and ability to detect low levels of viral DNA, particularly in acute infections. The more targeted application of PCR, as described in Table 1, likely contributes to its higher specificity, capturing cases that may not be as easily detected by serological methods such as the IgM Capture Assay. In 2024, the data show a significant increase in infections, with a total of 250 cases, including 50 pregnant women and five hospitalizations. The rising trend in infections suggests that the positivity rates for both IgM and PCR tests—2 and 4.5 times higher, respectively, than those observed in March 2017—could reflect the heightened sensitivity and specificity of these testing methods, as detailed in Table 1. The criteria for these diagnostic methods have guided our interpretation of the data, emphasizing the role of PCR in identifying acute infections and the broader application of IgM assays for detecting current or recent infections.

**Figure 2:**
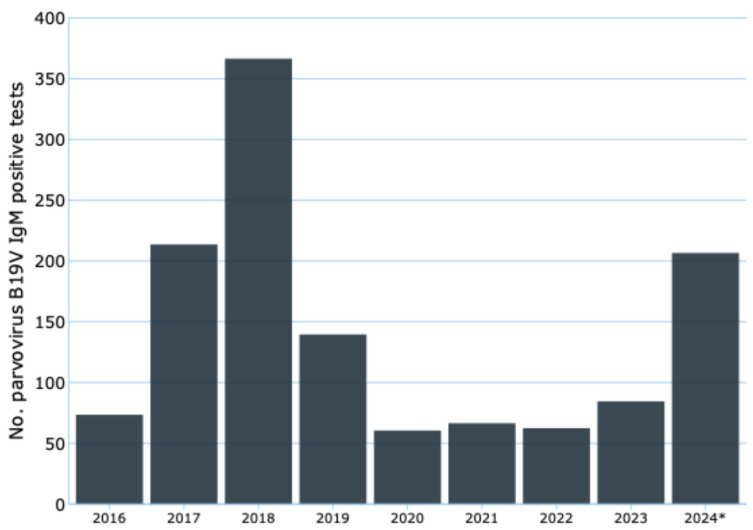
Parvovirus B19V IgM positive tests, 2016 to 2024 (* to end June 2024) [26,27].

Figure 2 shows the trend of positive tests for B19V IgM from 2016 to 2024 in Ireland, revealing a complex and dynamic picture. The graph shows marked annual variability, with a pronounced peak of 368 cases in 2018, followed by a rapid decline. This trend suggests a possible cyclicality in the incidence of the virus, potentially related to the accumulation of susceptible individuals in the population. The dramatic reduction observed between 2019 and 2020, from 140 to 59 cases, coincides with the onset of the COVID-19 pandemic, likely reflecting the impact of social distancing measures and changes in health priorities. The subsequent period from 2020 to 2024 shows a gradual recovery, with cases rising to 108 in 2024 (partial data through April), perhaps indicating a return to normalcy in testing and diagnosis practices or renewed viral circulation.

**Figure 3:**
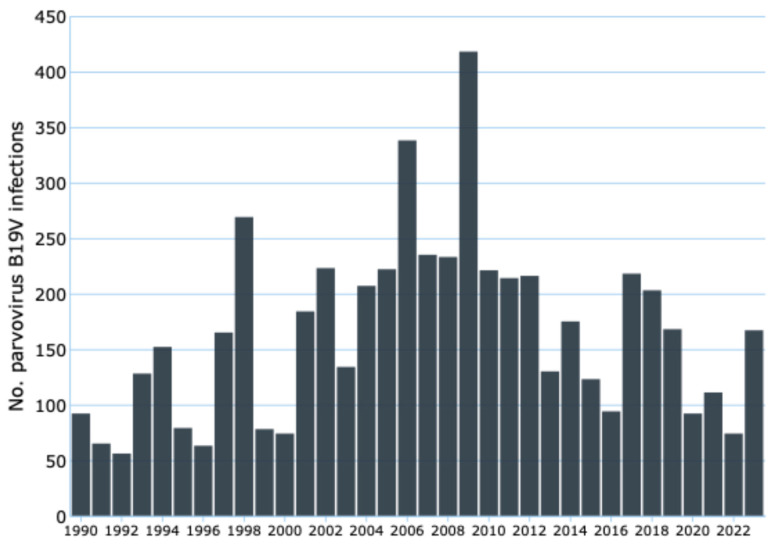
Absolute number of B19V infections reported annually to the Sentinel Surveillance system during the period from 1 January 1990 to 31 December 2023 in the Netherlands.

Based on Sentinel Surveillance data from the Netherlands [18], Figure 3 shows the trend in the number of cases from 1990 to 2023. The lowest value is recorded in 1992 with 56 cases, while the highest peak is observed in 2009 with 418 cases, with a variation of more than seven times between the lowest and highest values. 

Several phases can be identified in the trend of cases as follows: (i) the 1990s show a general upward trend, with a peak of 269 cases in 1998; (ii) the early 2000s see relative stability, followed by a marked increase from 2005 to 2009, reaching an absolute peak of 418 cases; (iii) from 2010 onward, there is a general downward trend, interrupted by some sporadic increases; (iv) the last few years, particularly 2020 to 2022, show relatively low numbers, possibly influenced by the COVID-19 pandemic, with a slight upswing in 2023 to 167 cases.

This increase has continued in the first few months of 2024, as reported by Municipal Public Health Services (GGDs), laboratories, the Netherlands Institute for Health Services Research (Nivel) [28], and the Sanquin national blood bank, which share information with National Institute for Public Health and the Environment (RIVM). Specifically, since February 2024, compared with previous years, general practitioners (GPs) have been seeing more children, especially in the 5–14 age group, with a viral illness with rash. In week 27, the number of children (0–4 years) coming to the GP with a viral illness with rash increased compared to the previous week from 129 to 145 per 100,000. In the age group of 5 to 14 years, the number of children with viral illness with rash decreased from 34 to 19 per 100,000. This is comparable to 2023.

**Figure 4:**
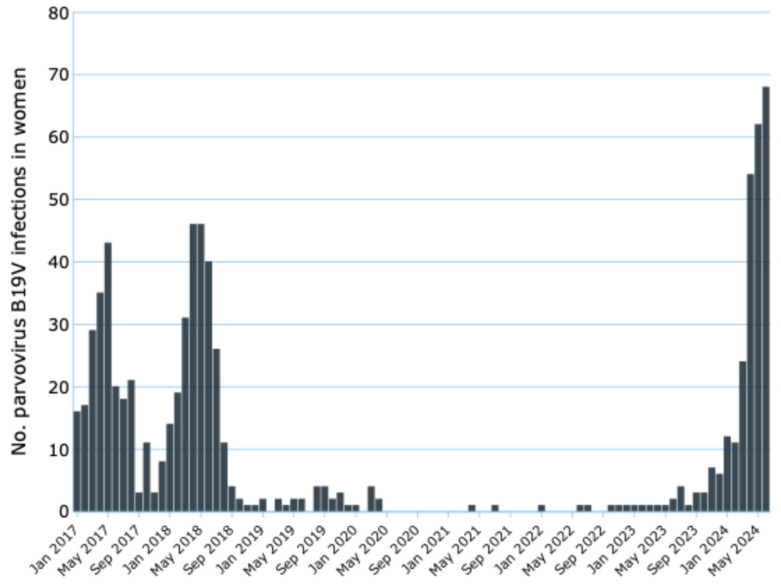
Monthly laboratory-confirmed reports of B19V infection in women aged 15 to 44 years in England, from January 2017 to June 2024 [29].

In England, the latest report published by the UK Health Security Agency [29] provided an analysis of the monthly trends in B19V infections from 2017 to 2024 (Figure 4). The analysis revealed a complex pattern characterized by significant seasonal and annual variations. Figure 4 illustrates these trends, showing the monthly number of reported B19V cases over the specified period. The chart shows a marked seasonal cyclicality, with recurring peaks tending to occur in the spring and summer months of each year. This cyclicality is particularly evident in 2017 and 2018, which show the highest values of the entire observed period. In addition, 2017 and 2018 show the most pronounced peaks, with values exceeding 40 cases per month in some months. These years appear to represent a period of major intensity for the condition under investigation. From 2019 onwards, there is a clear decrease in the number of cases, with much less pronounced peaks than in previous years.

This downward trend becomes even more evident from 2020 onwards, possibly influenced by the COVID-19 pandemic and related containment measures. The years 2020, 2021, and the first half of 2022 show extremely low values, with many months recording zero cases or numbers very close to zero. This could reflect either an actual decline in the incidence of the condition, or potential changes in testing or reporting practices during the pandemic period. 

Towards the end of 2022 and during 2023, there is a slight increase in the number of cases, suggesting a possible return to pre-pandemic patterns, albeit with numbers still being significantly lower than the 2017–2018 peaks. The beginning of 2024 shows a further increase, with values that, while still below historical peaks, are the highest observed since 2019. 

The Instituto de Salud Carlos III reported 305 cases from January to June 2024, marking a staggering 1029.63% increase compared to the 27 cases registered in the same period in 2023 [30]. This sharp rise is part of a broader trend seen over recent years, with case numbers fluctuating significantly. Specifically, there were 334 cases from 1 January to 7 July 2024, compared to 53 cases from 2 January to 24 December 2023, and 17 cases from 1 January to 18 December 2022. This pattern contrasts with the 25 cases reported in 2021, the 157 cases in 2020, and the notably higher 571 cases in 2019. The Andalusian government, on the other hand, registered a total of 1344 cases between January and May 2024, 253.6% more than in the same period in 2023 when 380 cases were registered [31]. By provinces, Almeria registered a total of 105 cases between January and June 2024, compared to 44 in the same period in 2023, which means 138.6% more; Cadiz had 233 cases in 2024, and 72 in 2023 (+223.6%); Cordoba had 110 cases in 2024, and 33 in 2023 (+233.3%); Granada had 304 cases in 2024, and 40 in 2023 (+660%); Huelva had 10 cases in 2024, and 12 cases in 2023 (−16.6%); Jaén had 150 cases in 2024, and 14 in 2023 (+971.4%); and Seville had 151 cases in 2024, and 79 in 2023 (+91.14%), according to the Junta. Other Member States reporting information through EpiPulse [10] include Latvia, which recorded 58 cases (with a 56% positivity rate among tested cases) for the 2023–2024 season, compared to zero to six cases during the same period (December to March) over the previous five years. Of these 58 cases, 67% were children. Norway noted that testing activity was highest among adults aged 30–59 years, who also represented the highest number of positive cases, primarily detected through IgM and PCR tests. The positivity rate increased in late January 2024 but did not continue to rise in the following months. On 6 June 2024, Austria’s blood safety authorities reported increased B19V DNA positivity in blood and plasma pools, particularly compared to the same period the previous year. Despite parvovirus infection not being notifiable in Austria, the ECDC contacted SoHO-Net National Focal Points (NFPs) for blood on 22 April 2024 to inquire about B19V testing among blood donors and any observed increases in infections. Eighteen countries responded, most of which do not routinely test blood donors. However, by 6 May 2024, 10 countries (Finland, Hungary, Luxembourg, Lithuania, the Netherlands, Czechia, Denmark, France, Germany, and Slovakia) reported an increase in reactive B19V tests in donor populations during early 2024 compared to the same period in 2023. On 26 April 2024, Czechia reported a ten-fold increase in erythema infectiosum cases compared to 2023. Similarly, on 25 April 2024, Latvia reported an increase in B19V cases, noting 58 cases (56% positive test results) for the 2023–2024 season compared to 0 to 6 cases in previous years. Of these cases, 67% were children. Lastly, on 17 April 2024, Lithuania reported increased B19V detections in blood donor screenings across several counties. 

The recent absence of B19V has likely increased the number of susceptible individuals, implying that a large-scale re-entry could lead to significant outbreaks, particularly affecting pregnant women. Post-pandemic data indicate a rise in B19V circulation, even during winter. Unexpectedly, the epidemic intensified in winter 2023–2024, with B19V DNA-positivity rates nearly doubling each month between September 2023 and January 2024. The rates from September 2023 to February 2024 were significantly higher than both the previous half-year and pre-COVID-19 periods [10,18].

Some researchers speculate that this unusual epidemiological pattern could be due to reduced herd immunity from low B19V circulation over the past two years. In April 2024, several European countries reported an increase in cases across all age groups, with children being the most affected [23,26,29,32,33,34,35]. Moreover, there was a notable rise in severe congenital infection complications, including miscarriages and neonatal deaths, in early 2024 compared to previous years.

## 4. Clinical Implication and Perspectives

**Figure 5:**
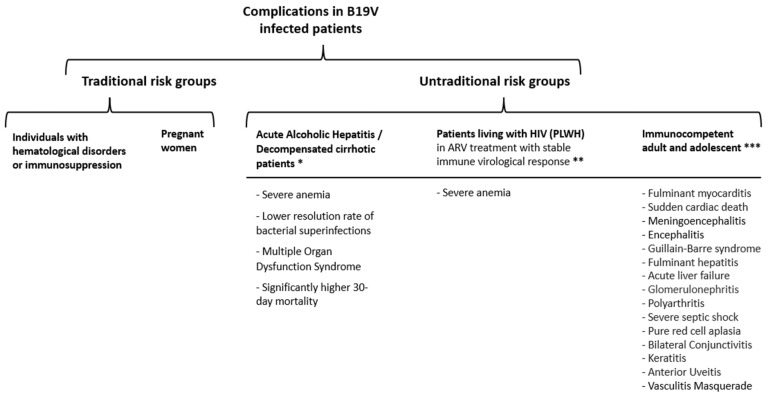
Severe complication of B19V infection reported from 2020 to 2024 in untraditional at-risk groups. Note: * [44,45]; ** [46]; *** [47,48,49,50,51,52,53,54,55,56,57].

Evolving epidemiological trends may alter the clinical presentation patterns of various viral infections. This raises a pertinent question: Does this hold true for B19V infection as well [36]? While B19V typically causes asymptomatic or mild infections in immunocompetent individuals, with rare fatalities, individuals with established risk factors, such as pregnant women and those with hematological disorders or immunosuppression, experience an elevated risk of severe disease and complications (Table 1 and Figure 5).

The recent surge in B19V cases across 14 EU/EEA countries, coupled with increased viral circulation and shifting epidemiological patterns, may signify a heightened exposure risk for a broader population segment, including those traditionally considered low risk, such as immunocompetent adults and adolescents. Although often perceived as a benign childhood illness, severe B19V infection is well documented in this demographic [37,38]. This underscores the need for a reassessment of risk stratification, even among immunocompetent adults and adolescents.

Indeed, B19V can precipitate severe clinical outcomes in individuals with undiagnosed underlying conditions or co-infections, who may not have been identified as high risk due to their presumed immunocompetence (Figure 5) [37,38]. Consequently, epidemiological shifts can elevate the risk of severe disease and complications, even in the absence of classical predisposing factors, highlighting the importance of recognizing and managing individuals with unrecognized vulnerabilities.

From an epidemiological perspective, the newly observed seasonality for B19V outbreaks has expanded from the late winter, spring, and early summer months to include the depths of winter. Consequently, several new variables affect the traditional model of transmission and clinical severity of the infection [39]. In particular, B19V spreads primarily through respiratory droplets, making close contact a significant risk factor. This is particularly true in winter, when low temperatures lead many people to share enclosed spaces for extended periods. Consequently, outbreaks are more common in settings like schools and daycare centers, but transmission can occur in households and other close-contact environments. Moreover, during outbreaks of respiratory illnesses, it is more likely for individuals to be infected with multiple viruses or other pathogens simultaneously. Simultaneous infection with other seasonal pathogens could potentially overwhelm the immune system, increasing the risk of severe B19V disease [40,41,42,43].

Focusing on immune response and antibody coverage, the primary difference between immunocompromised and immunocompetent subjects lies in the speed and effectiveness of the initial immune response. A healthy immune system acts quickly and decisively to combat viral infections, effectively reducing the severity and duration of the illness. In contrast, a compromised immune system may struggle to control the infection, leading to prolonged viral presence and potentially more severe outcomes. Additionally, while immunocompetent individuals typically excel at controlling viral replication and clearing the infection, immunocompromised individuals face a heightened risk of persistent, high-level viral replication due to their weakened immune response [58]. Finally, immunocompromised individuals are at a significantly higher risk of developing severe and potentially life-threatening complications from B19V [58]. However, several factors that are active during the progression of an outbreak—such as higher viral load, strain variations, and concurrent infections—can challenge even a healthy immune system, potentially leading to more severe symptoms [10]. The severity of B19V infection is also often linked to the immune system’s first encounter with the virus. Primary infection in adulthood can sometimes lead to more pronounced symptoms compared to childhood infections. Reinfection in immunocompetent individuals is usually mild or asymptomatic due to the presence of pre-existing immunity. While antibodies play a role in protection, cellular immunity, particularly cytotoxic T cells, is crucial in controlling and clearing B19V infection [59]. Any impairment in cellular immunity, even in otherwise healthy adults, could potentially increase the risk of severe disease. Studies indicate that a significant proportion of the adult population has been exposed to B19V, as evidenced by the presence of IgG antibodies [60]. This suggests a generally high level of immunity within the community. While IgG antibodies provide long-term protection against B19V, their levels may decline over extended periods, particularly in populations that have experienced primary infections many years ago. This waning immunity could potentially increase susceptibility to reinfection, especially during periods of heightened B19V activity or in the context of declining herd immunity. However, as mentioned earlier, reinfections are typically less severe than primary infections, possibly due to the partial immunity that persists despite the decline in antibody levels.

Concurrent infections can significantly impact the severity of B19V infection in immunocompetent adults due to a strain on the immune system [61,62]. Resource competition, related immune suppression, and inflammation and tissue damage can lead to a severe course [40,41,42,43,63]. When the body is fighting off multiple infections simultaneously, the immune system’s resources are divided. This means that there might not be enough immune cells and antibodies readily available to effectively target and eliminate both the B19V and the other concurrent infection. Some infections can temporarily suppress the entire immune system. This suppression can create a window of opportunity for B19V to replicate more aggressively, potentially leading to a higher viral load and more pronounced symptoms. Finally, concurrent infections can lead to increased inflammation and tissue damage throughout the body. While this is a natural part of the immune response, excessive or prolonged inflammation can exacerbate the symptoms of B19V infection and delay recovery [64,65,66,67,68,69,70,71,72,73].

Therefore, it is crucial to consider the possibility of concurrent infections when diagnosing and managing B19V in adults, even if they are generally healthy. 

From a clinical perspective, outbreaks can sometimes lead to a higher proportion of severe cases. Several factors might contribute to this as follows: Firstly, the increased exposure and viral load; secondly, viral strain variations; and, finally, individual immune response and concurrent infections. In particular, outbreaks naturally mean more people are infected and spreading the virus. This higher prevalence translates to a greater likelihood of exposure for everyone, including healthy individuals. Additionally, exposure to a higher “viral load” during an outbreak could potentially overwhelm even a robust immune system, leading to more severe symptoms [74,75,76,77]. Moreover, like many other viruses, B19V can have slight genetic variations between strains. Some strains might be more efficient at replicating or evading the immune system, potentially leading to more severe disease. During outbreaks, there is a higher chance of encountering a wider variety of strains, including those with a greater potential for causing severe illness. It remains to be shown whether there are novel clinical manifestations associated with different genotype infections, and the topic is currently under evaluation [78,79]. Regarding host immune response, even within the category of “immunocompetent,” there is a spectrum of immune system strength and responsiveness. Factors like genetics, stress, sleep, and nutrition can all subtly influence how effectively an individual’s immune system responds to a particular pathogen [80,81,82]. It is possible that during outbreaks, individuals who might have otherwise experienced a mild case of B19V could experience more severe symptoms due to subtle variations in their immune response at that time. Finally, as previously reported, outbreaks of respiratory illnesses often involve multiple viruses circulating simultaneously. It is not uncommon for someone to contract B19V alongside a cold, flu, or other respiratory infections [61]. 

These concurrent infections can strain the immune system, diverting resources and potentially hindering the body’s ability to effectively combat B19V. As a result, even individuals who are otherwise healthy may experience a more severe course of illness. Recognizing the impact of concurrent infections is crucial, as it allows healthcare providers to be more vigilant in monitoring and managing potentially severe cases during outbreaks, even in patients without known risk factors. In conclusion, although B19V is typically mild in healthy adults, studies have shown that, during outbreaks, the increased exposure to the virus and the higher likelihood of concurrent infections can significantly strain the immune system, leading to a more severe disease course. Additionally, the often subtle and nonspecific presentation of B19V in adults can further complicate a timely diagnosis, as noted in previous epidemiological studies [83]. These factors—heightened exposure, potential immune system compromise due to concurrent infections, and diagnostic challenges—combine to elevate the risk of more severe cases during outbreaks, even in individuals who might not otherwise be considered at high risk.

## 5. Current Diagnostic Challenges

The diagnosis of B19V infection involves a combination of cytopathology, molecular detection techniques, and serological tests, each applied based on specific diagnostic criteria (Table 2). In cytopathology, the presence of giant pronormoblasts in the bone marrow (BM) or peripheral blood is suggestive of B19V infection. However, the absence of these cells does not rule out infection, especially in patients with human immunodeficiency virus (HIV) or other chronic infections. Several methods are available for detecting B19V, including electron microscopy (EM), antigen enzyme-linked immunosorbent assay (ELISA), hemagglutination, direct hybridization, and polymerase chain reaction (PCR) (Table 2).

Electron Microscopy (EM) can detect B19V in serum, but its limited sensitivity and availability make it less common in modern diagnostics. Similarly, ELISA is employed to detect B19V antigens in serum, although it is increasingly supplanted by more sensitive DNA-based methods like Polymerase Chain Reaction (PCR). Hemagglutination, which detects B19V through the agglutination of red blood cells, is another method that is rarely used today due to its lower sensitivity and specificity. Direct Hybridization, often performed as a slot or dot blot, offers a more targeted approach by using labeled probes to isolate viral DNA from clinical specimens. This method is sufficiently sensitive to detect B19V in acute conditions like transient aplastic crisis and pure red cell aplasia, particularly in immunosuppressed patients. However, it may not detect lower levels of viremia. The advent of PCR has significantly enhanced the detection of B19V DNA, offering high sensitivity in both serum and tissue samples. PCR is particularly valuable for diagnosing acute infections and for monitoring chronic infections in immunocompromised patients, where detecting low levels of viral DNA is crucial. Serological tests complement these molecular methods by detecting B19V-specific antibodies, which are critical for diagnosing the virus in immunocompetent individuals presenting with erythema infectiosum or B19V-induced arthropathy. IgM Capture Assays are particularly reliable for identifying current or recent infections, as IgM antibodies typically appear 3–10 days after the onset of clinical illness and remain detectable for 2–3 months. In contrast, IgG antibodies, which appear 7–14 days after infection and persist for life, are more useful for indicating past infection or immunity. IgG detection is also helpful in assessing seroconversion in immunocompromised patients. Additionally, NS1-specific antibodies, especially NS1-specific IgG, are considered in cases of persistent infection or arthritis, as their late appearance in infection may indicate prolonged viremia and involvement of non-erythroid cells.

## 6. A Call for Proactive Clinical Management

Heightened awareness and prompt diagnostics are crucial for managing potential B19V outbreaks to prevent unexpected severe cases in immunocompetent adolescents and adults [57]. This vigilance is also crucial for other at-risk populations, including those who are pregnant, individuals with frailty due to advanced age or comorbid conditions, and those with primary or secondary immunodeficient

In light of these considerations, a reassessment of clinical strategies during periods of heightened B19V activity is warranted. We advocate for a more inclusive approach that moves away from the traditional dichotomy of distinct risk groups versus a protected immunocompetent population. It is important to recognize that certain risk factors may predispose individuals to more severe B19V infection. These risk factors include recent exposure to children, who are often asymptomatic carriers of the virus, underlying medical conditions such as chronic hemolytic anemia or immunosuppression, and the use of medications that may impair the immune response. Understanding these factors is crucial in identifying patients who may be at higher risk for complications. Specifically, we recommend the following: (a) Maintain a high index of suspicion for B19V infection in all patients presenting with relevant symptoms, regardless of immune status; (b) Conduct thorough history-taking to identify potential risk factors, including recent exposure to children, underlying medical conditions, and current medications; (c) Consider broadening testing criteria to include individuals with unexplained hematological abnormalities, persistent arthralgia, or suggestive cardiac, hepatic, or neurological symptoms, particularly during periods of heightened B19V activity; (d) Enhanced public health surveillance efforts are crucial to monitor B19V epidemiology, identify potential outbreaks promptly, and inform targeted public health interventions.

By acknowledging the potential for severe B19V infection in all individuals, regardless of perceived immune status, and adopting proactive diagnostic and management strategies, we can mitigate the risk of serious complications and improve patient outcomes across the board.

## Figures and Tables

**Table 1:** Clinical presentation of B19V.

	Traditional Risk Factors		Immuno Competent Individuals
	Immuno Compromised Individuals	Pregnancy	
Clinical presentation	Chronic Anemia Aplastic Crisis Other Complications: Myocarditis, encephalitis, and hepatitis	Fetal Hydrops Miscarriage Stillbirth	Erythema Infectiosum o “Slapped cheek” rash o Fever, headache, malaise. o Reticular rash (trunk and limbs) Arthralgia and Arthritis Less common: fatigue, sore throat, diarrhea, and conjunctivitis.

**Table 2:** Diagnostic Methods for B19V.

Diagnostic Method	Description	Key Findings	Clinical Implication
Cytopathology	Identification of giant pronormoblasts in BM or peripheral blood.	Suggestive of B19V infection, but not definitive. Often absent in HIV or chronic infections.	Should be used in conjunction with other diagnostic methods.
Electron Microscopy (EM)	Detection of B19V in serum.	Limited sensitivity and availability; not commonly used.	Supplementary method; not routinely used in diagnosis.
Antigen Enzyme-Linked Immunosorbent Assay (ELISA)	Detection of B19V antigens in serum.	Useful for detecting B19V antigens; less commonly used than DNA-based methods.	Can support diagnosis, especially in conjunction with other tests.
Hemagglutination	Detection of B19V through agglutination of red blood cells.	Rarely used in modern diagnostics.	Supplementary method.
Direct Hybridization (Slot/Dot Blot)	Isolation of B19V DNA using labeled probes in clinical specimens.	Sensitive for detecting B19V DNA in acute and chronic infections; detection limit: ~105 genome copies/mL.	Effective for detecting B19V in acute cases and immunosuppressed patients.
Polymerase Chain Reaction (PCR)	Amplification and detection of B19V DNA in serum and tissue samples.	Highly sensitive; detects low levels of viral DNA.	Primary method for diagnosing acute infection; essential for immunocompromised patients.
Quantitative PCR (qPCR)	Measures viral load in clinical samples.	Quantifies viral DNA; useful for monitoring infection severity and treatment response.	Under investigation for blood product screening; important for managing severe cases.
IgM Capture Assay	Detection of B19V-specific IgM antibodies.	Detects current or recent infection; IgM appears 3–10 days after onset and remains for 2–3 months.	Reliable for diagnosing recent infection in immunocompetent individuals.
IgG Capture/Indirect Assay	Detection of B19V-specific IgG antibodies.	IgG appears 7–14 days after onset and persists for life.	Useful for determining past infection or immunity; not definitive for acute infection.
NS1-Specific Antibody Detection	Detection of antibodies against the nonstructural protein NS1.	NS1-specific IgG appears late (>6 weeks); may indicate persistent infection or arthritis.	Can help exclude recent infections; controversial role in indicating persistent infection.
